# Proteomic and Phosphoproteomic Analysis in Tobacco Mosaic Virus-Infected Tobacco (*Nicotiana tabacum*)

**DOI:** 10.3390/biom9020039

**Published:** 2019-01-23

**Authors:** Zi-Shu Lu, Qian-Si Chen, Qing-Xia Zheng, Juan-Juan Shen, Zhao-Peng Luo, Kai Fan, Sheng-Hao Xu, Qi Shen, Ping-Ping Liu

**Affiliations:** 1 College of Chemistry and Molecular Engineering, Zhengzhou University, Zhengzhou 450000, China; luzishu199422@163.com (Z.-S.L.); m18339830149@163.com (J.-J.S.); 2 Zhengzhou Tobacco Research Institute of CNTC, Zhengzhou 450000, China; chenqs@ztri.com.cn (Q.-S.C.); zhengqingxia916@126.com (Q.-X.Z.); luozhaopeng@163.com (Z.-P.L.); fankaiuk@163.com (K.F.); 3 Key Laboratory of Sensor Analysis of Tumor Marker, Ministry of Education, College of Chemistry and Molecular Engineering, Qingdao University of Science and Technology, Qingdao 266042, China; xushenghao@qust.edu.cn

**Keywords:** tobacco, tobacco mosaic virus (TMV), phosphoproteomic, proteomic, isobaric tags for relative and absolute quantitation (iTRAQ) labeling, nano-LC–MS/MS

## Abstract

Tobacco mosaic virus (TMV) is a common source of biological stress that significantly affects plant growth and development. It is also useful as a model in studies designed to clarify the mechanisms involved in plant viral disease. Plant responses to abiotic stress were recently reported to be regulated by complex mechanisms at the post-translational modification (PTM) level. Protein phosphorylation is one of the most widespread and major PTMs in organisms. Using immobilized metal ion affinity chromatography (IMAC) enrichment, high-pH C18 chromatography fraction, and high-accuracy mass spectrometry (MS), a set of proteins and phosphopeptides in both TMV-infected tobacco and control tobacco were identified. A total of 4905 proteins and 3998 phosphopeptides with 3063 phosphorylation sites were identified. These 3998 phosphopeptides were assigned to 1311 phosphoproteins, as some proteins carried multiple phosphorylation sites. Among them, 530 proteins and 337 phosphopeptides corresponding to 277 phosphoproteins differed between the two groups. There were 43 upregulated phosphoproteins, including phosphoglycerate kinase, pyruvate phosphate dikinase, protein phosphatase 2C, and serine/threonine protein kinase. To the best of our knowledge, this is the first phosphoproteomic analysis of leaves from a tobacco cultivar, K326. The results of this study advance our understanding of tobacco development and TMV action at the protein phosphorylation level.

## 1. Introduction

Tobacco mosaic virus (TMV) is a major source of biotic stress that significantly affects tobacco growth and development. It is highly harmful to tobacco, tomato, rape, potato, and other crops, causing serious damage and large economic losses [1]. TMV is one of the most intensively studied model organisms in the field of cell-to-cell movement. Once a plant is infected with TMV, it remains infected throughout its growth, and appropriate environmental conditions can spread TMV infection over wide areas. Pesticides are a major method used to defend plants against TMV; however, they are not good for the environment and are not very effective. Substantial research on TMV prevention was reported [2,3,4,5], but the desired effect is yet to be achieved. Therefore, novel mechanisms and technologies for preventing this viral infection are critically needed.

Tobacco is a major crop that is widely cultivated throughout the world for its economic value. Tobacco is also a typical Solanaceae model plant and is easily infected by TMV [6]. For this reason, research on the resistance mechanism of TMV-infected tobacco is not only of biological interest, but also important in agriculture. There are many reports about drought in plants, but only a few studies were conducted on tobacco gene expression profiles, and there are no previous studies on tobacco protein phosphorylation. Our analysis of the tobacco dataset revealed features that are consistent with those of other plant phosphoproteomes, such as the relative proportions of phosphorylated Ser, Thr, and Tyr residues, and the phosphorylation events associated with viral defense.

Post-translational modification (PTM), one of the best-studied types of modification, is involved in the regulation of a large number of processes, including homeostasis, transcription, translation, protein degradation, metabolism, and cellular signaling and communication [7]. More than 200 types of PTMs are estimated to exist in proteins. Protein phosphorylation is one of the most widespread and major PTMs in organisms. This modification is highly accurate and reversible and modulated by a series of kinases and phosphatases as part of a signaling cascade [8]. 

Some disease resistance-related proteins are also believed to be regulated by phosphorylation, and tremendous progress was achieved in understanding the importance of protein phosphorylation in plant defense signaling [9]. Increasing evidence shows that phosphorylation might be a significant signal underlying the changes observed during TMV infection in plants. The disease response and tolerance mechanisms of plants tend to involve hormone signaling and protein degradation. Some reports concluded that the abscisic acid (ABA)-induced protein phosphatase 2C (PP2C) signaling pathway is related to drought response and defense [10,11]. The chloroplast was believed to be involved in viral infection in plants for approximately 70 years [12], and it is a common target of plant viruses for viral pathogenesis or propagation.

Clarifying the mechanism of plant phosphoprotein tolerance to TMV infection is critical. Previous studies on plant gene expression changes applied comparative transcriptomics or proteomics methods [13]. Recently, a few transcriptome and proteome analyses were performed to examine the defense mechanism against TMV infection mediated by a plant-originated biopesticide in tobacco, and the results identified the differential expression of some genes involved in salicylic acid-mediated pathways and secondary metabolism [14,15,16]. Meanwhile, few phosphoproteomic studies, especially on tobacco, were carried out to date [17]. Our study identified many proteins that exhibited significant changes in phosphorylation level (SCPL) with high-fold changes between TMV-infected tobacco and control tobacco, which strongly suggests that early events in viral and plant interaction are largely mediated by alterations in protein PTMs, such as phosphorylation.

In this study, we explored the mechanism via which the PTM level of K326 (*Nicotiana tabacum*) is related to resistance to TMV infection. The tobacco system provides an ideal model for studying plant–viral cross-talk due to the numerous studies of TMV infection resistance in plants. Here, we conducted the enrichment of phosphopeptides and phosphorylation sites in tobacco on a large scale before and after 48 h of TMV infection. Using isobaric tags for relative and absolute quantitation (iTRAQ) labeling and immobilized metal ion affinity chromatography (IMAC) enrichment, 1317 phosphoproteins with 3063 phosphorylation sites and 530 specifically expressed proteins (SEPs) were identified. The iTRAQ quantification of phosphopeptides was performed using high-accuracy triple time-of-flight (TTOF) 5600+ (AB SCIEX, Redwood City, CA, USA) combined with pilot software. Gene Ontology (GO) and Kyoto Encyclopedia of Genes and Genomes (KEGG) pathway analyses of differentially accumulated phosphoproteins were performed to suggest potential resistance-related phosphoproteins. The results of a phosphoproteomic analysis of tobacco samples indicated that the phosphorylation levels changed significantly after 48 h of cultivation in TMV-infected tobacco K326. The analysis results reported herein provide the first global survey of the phosphoproteomic profile of tobacco. Our results also show some phosphoproteins located on the chloroplast, such as pyruvate phosphate dikinase, chlorophyll a/b binding protein 21, and protein TIC 62, suggesting that the chloroplast and its components can play central roles in plant defense against viruses.

These results may help elucidate the physiological functions underlying serine/threonine/tyrosine (Ser/Thr/Tyr) phosphorylation, as well as help construct signaling networks in tobacco and analyze the related biological processes. They will also be helpful for further exploration of the causes of resistance to TMV infection in tobacco. 

## 2. Materials and Methods

### 2.1. Plant Growing, Viral Inoculation, and Taking of Samples

Eight samples of the tobacco cultivar K326 were divided into two equal groups, namely the treatment (T) and control (C) groups. After two months of growth in quartz sand with nutritive water, the T group was inoculated with TMV as described in previous reports [18,19,20]. TMV was maintained in our laboratory and multiplied in tobacco. TMV was extracted from infected tobacco leaves by homogenization in 1% K_2_HPO_4_ solution (pH 6.8) (1 g/mL), and the supernatant of the extract was used for mechanical inoculation after centrifugation at 6000× *g* for 5 min. The fifth true leaves of T group tobacco plants were used for TMV inoculation. Inoculation was performed by gently rubbing the leaf surface using carborundum (silicon carbide) with a viral suspension at 22 °C. The T and C groups were placed in greenhouses. After 48 h, the two groups of samples collected were immediately frozen in liquid nitrogen and kept at −80 °C until used for protein extraction.

### 2.2. Quantitative Reverse-Transcription Polymerase Chain Reaction (RT-PCR) Analysis

Total ribonucleic acid (RNA) was isolated from TMV-infected tobacco leaves at 48 h and 0 h after inoculation using TRIzol (GENE ANSWER, SuperPure Plantpoly RNA Kit, Beijing, China) according to the instructions. The RNA concentration was measured with a NanoDrop 2100 spectrophotometer (Thermo Scientific, Waltham, MA, USA). One microgram of total RNA was used for reverse transcription with the First-Strand complementary DNA (cDNA) Synthesis Kit (Aidlab, Beijing, China). For real-time quantitative RT-PCR, the primers used were 5′–CTGTCGCCGAATCGGATTCG–3′ (upstream primer) and 5′–CAGGACCAGAGGTCCAAACCAAAC–3′ (downstream primer) (Supplemental S1, Supplementary Materials). Quantitative PCR was performed in 96-well plates with a total reaction volume of 10 μL (5 μL of 2× Taq MasterMix (Dye) (Aidlab), 1 μL of cDNA, 1 μL of primers, and 3 μL of water) on the LightCycler^®^ 96 real-time PCR detection system (Roche, Basel, Switzerland). The temperature settings were as follows: 95 °C for 15 s, followed by 40 cycles of 95 °C for 15 s, and annealing at 60 °C for 30 s. TMV expression was assessed by evaluating the threshold cycle (CT) values. The relative expression level was calculated using the 2-ΔΔCT method [21]. The experiment was performed on eight samples.

### 2.3. Sample Preparation and Instrument Settings for GC–MS Analysis

Gas chromatography/mass spectrometry (GC–MS) analysis was carried out according to Reference [22] with some modification. The eight samples were prepared for GC–MS as follows: the freeze-dried samples were ground to a uniform powder, filtered through a 40-mesh sieve, and stored at −80 °C until used for metabolic experiments.

Approximately 20 mg of each samples was added to 1.5 mL of isopropanol/acetonitrile/water (3/3/2, *v*/*v*/*v*) with 2 mg/mL tridecanoic acid as an internal standard. They were sonicated for 1 h, and then 500 μL of supernatant was collected after centrifugation at 14,000 rpm for 10 min. The supernatant was dried under nitrogen flow on an N-EVAP Nitrogen Evaporator (Organomation Associates, Berlin, MA, USA). Then, we added 100 μL of methoxyamine pyridine solution (20 mg/mL) (Merck KGaA, Darmstadt, Germany), incubated the samples for 90 min at 37 °C, added 100 μL of methyl-trimethyl-silyl-trifluoroacetamide (MSTFA) (Merck) to samples, before further incubating the samples for 60 min at 60 °C.

GC–MS analysis was performed on an Agilent 7683B series injector (Agilent, Santa Clara, CA, USA) coupled to an Agilent 6890N series gas chromatograph system and a 5975 mass selective detector (MSD) (Agilent) with an Agilent DB-5MS column (0.25 μm, 0.25 mm × 30 m, (Agilent)). The injection temperature was set to 300 °C. The injection volume was 1 μL with a 10:1 split ratio. The column temperature was 70 °C for the first 4 min, and it was increased at 5 °C/min to 310 °C for 15 min. The column flow was 1.2 mL/min. The mass scanning scope was set to 33–600 *m*/*z* in full scan mode, and 90 chromatographic peaks of 25 groups were set for selected ion monitoring. The solvent cut-off time was 5.0 min and the scan speed was 2.59 scan·s^−1^. The temperatures of the ion source and the interface were adjusted to 230 and 280 °C, respectively. The electron impact (EI) model was set to 70 eV, and the detector voltage was maintained at 1.2 kV.

### 2.4. Protein Extraction and Digestion

Plant protein extraction is the first step in proteomic studies. Eight plant material samples were harvested in four biological replicates of two groups.

As previously described by Ming et al. [23], approximately 1.5 g of fresh leaves from each biological replicate was manually ground to a fine powder in liquid nitrogen. The ground powder was then suspended in 8 mL of sodium dodecylsulfate (SDS) buffer (30% sucrose, 2% SDS), 20 mM dl-dithiothreitol (DTT), 100 mM Tris(hydroxymethyl)aminomethane hydrochloride (Tris–HCl, pH 8.0) with PhosSTOP Phosphatase Inhibitor Cocktail (one tablet/10 mL; PhosSTOP, Roche) and Complete Protease Inhibitor Cocktail (one tablet/10 mL; EDTA free, Roche) in a 50-mL centrifuge tube and vortexed strongly until precipitation occurred, followed by the addition of an equal volume of Tris-saturated phenol. The mixture was thoroughly vortexed for 10 min at 4 °C, and the phenol phase was separated by centrifugation at 15,000× *g* and 4 °C for 15 min. The top phenol phase was transferred to a fresh 50-mL centrifuge tube, and five volumes of cold methanol plus 100 mM ammonium acetate was added. The mixture was then incubated overnight at 20 °C. The proteins were finally precipitated by centrifugation (10 min, 15,000× *g* at 4 °C). The precipitated proteins were washed twice each with cold methanol and acetone, separately, then dried and resuspended in 2 mL of a solution containing 8 M urea and 50 mM ammonium bicarbonate. Finally, the protein mixtures were harvested by centrifugation. Using the Pierce^TM^ bicinchoninic acid (BCA) Protein Assay Kit (Thermo Scientific) containing bovine serum albumin (BSA) (2 mg/mL) as the standard, the protein content was measured.

For protein digestion, 2 mL of a 5 mg protein mixture was mixed with 5 mM DTT for 30 min at 56 °C. Then, the sample was alkylated by incubating in 40 mM iodoacetamide for 40 min in the dark. After the addition of 10 mM DTT for 40 min in the dark, trypsin was added to the protein sample at a 1:50 ratio (trypsin/protein, *w*/*w*). The protein sample was digested by incubation at 37 °C on a rocking shaker overnight. Sample desalination was performed by HLB solid-phase extraction (SPE) (Agilent, USA), and the sample was then washed twice with 3 mL of 1% trifluoroacetic acid (TFA) and eluted in 2 mL of 80% acetonitrile (ACN) and 0.1% TFA. The elution was dried under vacuum and stored at −80 °C.

### 2.5. iTRAQ Labeling Quantification and Fractionation

The iTRAQ labeling protocol was performed as described in the company manual. The tryptic peptides of each sample (100 μg) were incubated with the iTRAQ^®^ Reagent-8Plex Multiplex Kit (AB SCIEX) (113, 114, 115, and 116 for T; 117, 118, 119, and 121 for C) for 3 h at room temperature. After labeling and quenching, the samples were combined and lyophilized before redissolving in 100 μL of buffer A (pH 10, 0.05 mol/L ammonium formate in water). The labeled peptides were fractionated using a high-performance liquid chromatography (HPLC) system (Agilent 1260) connected to an Agela Durashell C18 (5 μm, 100A, 4.6 × 250 mm) (AB SCIEX). Peptides were eluted from the C18 with 80% ACN/20 mM ammonium formate (pH 10). Peptide separation was performed using a nonlinear 50-min gradient: from 0 to 5 min, 95% solvent A (20 mM ammonium formate, pH 10); from 5 min to 15 min, the ratio was changed to 35% solvent B (20 mM ammonium formate, 80% ACN, pH 10); from 15 min to 35 min, the ratio was changed to 90% solvent B; and from 35 to 45 min, 90% solvent B was used. At 50 min, the concentration of solvent B was 5%. The flow rate was 500 μL/min. Fractions were collected every minute from 3 min to 43 min. Each fraction was lyophilized and then desalted using a Ziptip (Merck Millipore, Germany. The eluted peptides from each fraction were divided into five aliquots, lyophilized, and stored at 20 °C.

### 2.6. IMAC Enrichment of Phosphopeptides

The dried peptides were enriched using the IMAC method. The Ti^4+^-IMAC material (Dalian Institute of Chemical Physics, Chinese Academy of Sciences) was prepared and used as described by Zhou [24]. The enrichment procedures for the five fractions were as follows: firstly, the loading buffer (80% ACN, 6% TFA), washing buffer A (50% ACN, 6% TFA, 200 mM NaCl), washing buffer B (30% ACN, 0.1% TFA), and elution buffer (10% ammonia solution, *w*/*w*) were prepared. Next, the Ti^4+^-IMAC material was mixed with each fraction in loading buffer with a Ti^4+^-IMAC: protein ratio of 10:1 (*w*/*w*). After vigorous shaking for 40 min at room temperature, the mixture was centrifuged (15,000 rpm, 6 min), and the supernatant was removed. The precipitate was sequentially washed with washing buffers A and B, followed by vigorous shaking for 30 min, centrifugation, and removal of the supernatant. Finally, the precipitate was incubated twice in 200 μL of elution buffer with shaking for 15 min, and the supernatant was collected and combined after centrifugation (20,000 rpm, 10 min). The combined supernatant was centrifuged (20,000 rpm, 5 min) again, and the resulting supernatant was collected and lyophilized for LC–MS/MS analysis.

### 2.7. Nano-LC–MS/MS Analysis

Four biological replicates of each sample were performed independently for protein and phosphopeptide identification using LC–MS/MS. LC–MS analysis was performed on a Nanospray III source and a TripleTOF 5600+ (AB SCIEX) mass spectrometer in information-dependent acquisition mode [25].

The phosphopeptide fractions were resuspended in 0.1% formic acid (FA) and loaded separately into an Ekspert^TM^ nanoLC 415 (AB SCIEX) with a nanoLC trap (ChromXP C18-CL 3 μm 120 Å, 350 μm × 0.5 mm), before being washed for 15 min with a flow rate of 2 μL/min. Then, the pump flow was split to obtain a flow rate of 300 nL/min on the nanoLC trap and the nanoLC column (75 μm × 15 cm, 3C18-CL-120, 3 μm, 120 Å) was used for MS analysis. The mobile phases consisted of 0.1% FA and water (A), and 0.1% FA and ACN (B). A nonlinear gradient of 5% to 8% B for 0.5 min, 8% to 22% B for 40 min, 22% to 30% B for 40 min, 30% to 50% B for 15 min, 50% to 80% B for 10 min, 80% B for 1 min, 80% to 5% B in 112 min, and 5% B for 8 min was employed. The mass tolerance was set to 50 mDa, and the detection limit was set to 100 cps. The spray voltage was set to 2.5 kV, and the temperature of the heated capillary was set to 150 °C.

For data acquisition, each MS spectrum was acquired across the mass range of 350–1500 *m*/*z* in high-resolution mode (>25,000) using a 250-ms accumulation time. A maximum of 30 precursors per cycle and a mass range of 350–1250 *m*/*z* were chosen for fragmentation from each MS/MS spectrum, with 50-ms minimum accumulation time for each precursor and dynamic exclusion for 20 s. The collision energy was set to use iTRAQ reagent energy. The precursor ion mass tolerance was 7 ppm.

The raw files were processed using ProteinPilot software and were then searched against the K326 database [26]. The search parameters were as follows: the sample type was set to iTRAQ 8plex, and the enzyme setting was Trypsin. The Cys Alkylation was Iodoacetamide, and the Special Factors setting was selected to emphasize phosphorylation. The false discovery rate (FDR) was set to <1.0% for the identification of both peptides and proteins, and the minimum peptide length was set to 7. For the TripleTOF 5600+ data on phosphopeptides, the results of the searched data files were processed into “.txt” files for analysis.

### 2.8. Bioinformatics Analysis

The identified proteins were annotated and classified according to GO functions using Web Gene Ontology Annotation Plot (WEGO, Version: 2.0 in 2018, The Beijing Genomics Institute (BGI), Shenzhen, China) [27]. The input file format selected was the WEGO native format, and the three GO levels were selected after clicking to upload the raw file.

The Motif-X algorithm (Version: 1.2 10.05.06, Harvard University, Cambridge, MA, USA) was used to extract significantly enriched amino-acid motifs surrounding the identified phosphorylation sites. The sequence window was limited to 13 amino acids, and foreground peptides were pre-aligned with the phosphorylation sites as centers. *Arabidopsis* proteome data were used as the background database. The occurrence threshold was set at a minimum of 20 peptides, and the *p*-value threshold was set at <10^−6^.

The Search Tool for the Retrieval of Interacting Genes/Proteins (STRING, Version: 10.5, Genome Campus, Hinxton, UK) database of physical and functional interactions was used to analyze the protein–protein interactions (PPIs) of the phosphoproteins identified in the current study. To improve the reliability of PPI analysis, the confidence score was set to a high confidence level (0.700).

## 3. Results

### 3.1. Real-Time PCR Analysis and Metabolomic Changes in TMV Tobacco

**Figure 1:**
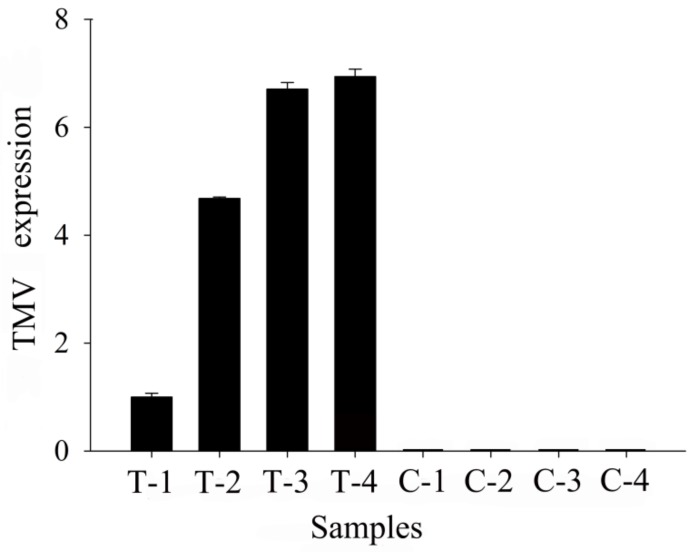
Tobacco mosaic virus (TMV) expression determined by qRT-PCR after 48 h of TMV infection.

To determine whether the T-group samples were infected with TMV, the TMV expression in the two groups was investigated after T-group inoculation with TMV. TMV is one of the most widely used models for understanding various aspects of pathogen resistance. To provide accurate data on the TMV expression in samples, the TMV expression was detected by qRT-PCR. The numbers T-1, T-2, T-3, and T-4, and C-1, C-2, C-3, and C-4 represent the four samples under the treatment and control conditions, respectively. The expression of TMV in the T group was very obvious, while the C group had no TMV expression. Thus, the results showed that the T-group samples were infected by TMV, whereas the controls were normal without infection (Figure 1).

The GC–MS analysis identified 120 differential components among 151 total detected components. The upregulated components included meso-erythritol, l-5-oxoproline, 3-ethylbutanoic acid, and arabinose, while the downregulated components included 1,2,3,6-tetrahydro-2,3′-bipyridine, l-glutamic acid, and 4-fluorobenzoic acid (Table S3, Supplementary Materials).

Erythritol occurs naturally in certain fruits, vegetables, and fermented foods. In plant cells, it is related to biofilm penetration [28] and oxidation resistance [29] because it is not easily degraded by enzymes. Glutamate can be produced from 5-oxoproline and then converted to glutamine [30]. After TMV infection, the contents of these components showed clear changes, which suggests that they are related to plant resistance to some extent.

### 3.2. Primary Proteome and Phosphoproteome Data on TMV-Infected Tobacco

**Figure 2:**
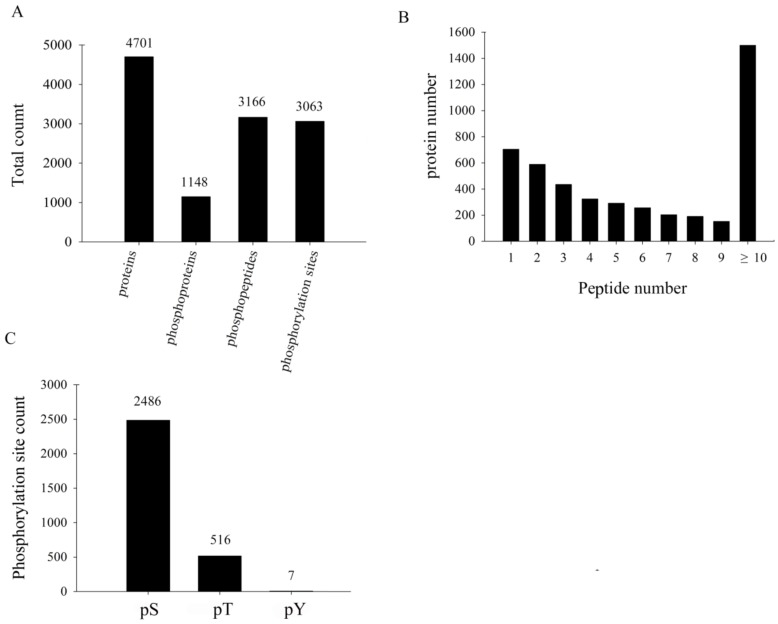
Overview of the identified proteins and phosphopeptides. (**A**) The numbers of identified proteins, phosphoproteins, phosphopeptides and phosphorylation sites (FDR <1%); (**B**) The distribution of the number of peptides defining each protein; (**C**) The numbers of phosphoserine (pS), phosphothreonine (pT) and phosphotyrosine (pY) residues in the phosphorylation sites.

The total proteins and phosphoproteins in the eight samples of the two groups (T and C) were explored by iTRAQ labeling (Figure S1, Supplementary Materials). The identified proteins were separated into new groups by aligning all proteins and grouping the proteins with the iTRAQ result. A total of 4905 proteins and 1317 iTRAQ phosphoproteins were detected. Among them, 4701 proteins with FDR <1% were identified as No-IMAC proteins. In addition, Ti^4+^-IMAC enrichment and LC–MS/MS analysis identified 3063 phosphorylation sites, representing 1148 phosphoproteins with FDR <1%, as shown in Figure 2A.

The distribution of the number of peptides defining each protein in the analysis without phospho-enrichment is shown in Figure 2B. Over 80% of the proteins included at least two peptides. In addition, the numbers of phosphoserine (pS), phosphothreonine (pT), and phosphotyrosine (pY) residues in the 3063 phosphorylation sites were 2486, 516, and 7, respectively (Figure 2C). The distribution of phosphorylation types in our study is consistent with other reports [31]. In addition, the serine/threonine protein kinase and serine/arginine-rich (SR) proteins were obviously upregulated in our study. They were related to serine; thus, the numbers of pS were the largest proportion.

### 3.3. SEP and SCPL Proteins

Master tables (Tables S1 and S2, Supplementary Materials) were generated that summarized all changed proteins and phosphoproteins. The master tables were useful for understanding the role of these proteins and phosphoproteins in the tolerance of K326 to TMV.

**Figure 3:**
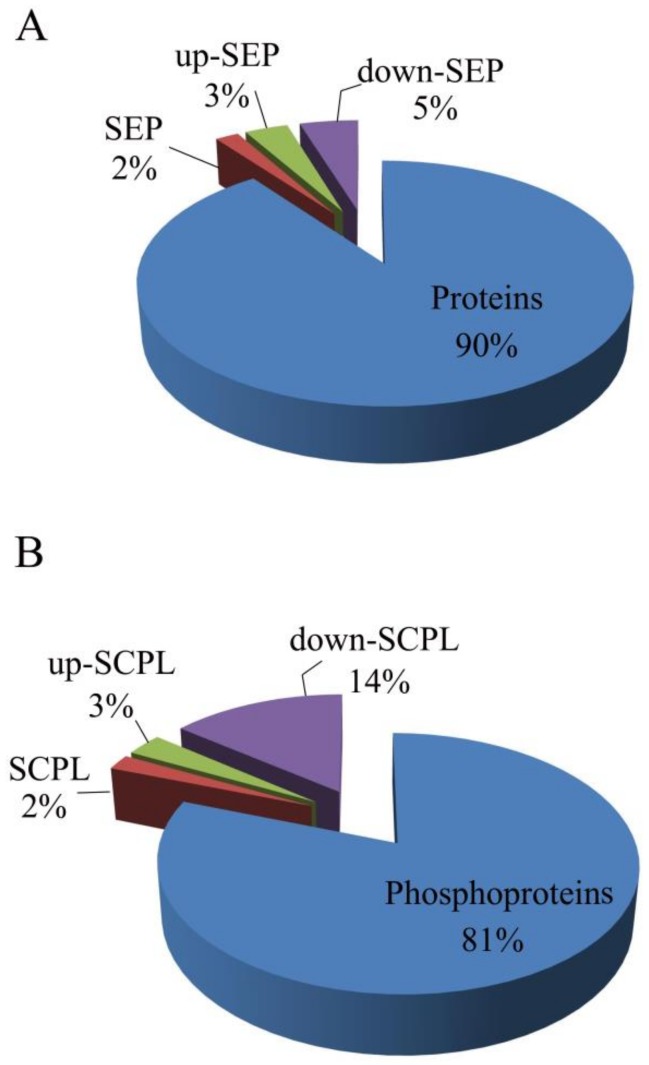
(**A**) Distribution of up-, mid-, and downregulated specifically expressed proteins (SEPs) in the identified proteins; (**B**) Distribution of up-, mid-, and downregulated significant changes in phosphorylation level (SCPL) proteins in the identified phosphoproteins.

**Figure 4:**
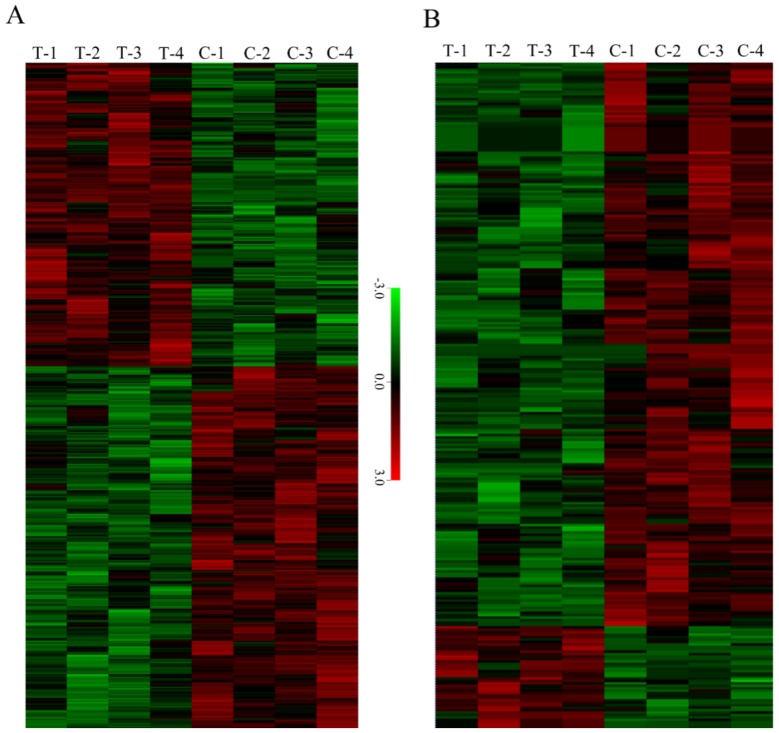
Hierarchical clustering of all of the differentially expressed protein (**A**) and phosphoprotein (**B**) profiles of tobacco leaves in control and TMV-infected plants.

To further study the different relationships of proteins and phosphoproteins in response to TMV infection in “T vs. C groups”, they were compared in two diagrams (Figure 3A,B). After TMV infection, in SEP, differential accumulation was observed for 183 upregulated proteins (*t*-test <0.05, fold-change >1.25), 248 downregulated proteins (*t*-test <0.05, fold-change <0.75) and mid-regulated proteins (*t*-test <0.05, 0.75 < fold-change < 1.25). More importantly, 43 phosphoproteins were upregulated, 29 phosphoproteins were mid-regulated, and 205 phosphoproteins were downregulated among SCPL proteins. In addition, clustering analysis (Figure 4A,B) indicated that the T group’s proteome and phosphoproteome were clearly distinguishable from those of group C. In SCPL proteins, the downregulated proteins were obviously more common than upregulated proteins, unlike the ratio seen for SEP. These specific TMV-responsive proteins and phosphoproteins might be crucial and valuable factors in the mechanism of tolerance to TMV infection.

### 3.4. GO Analysis

**Figure 5:**
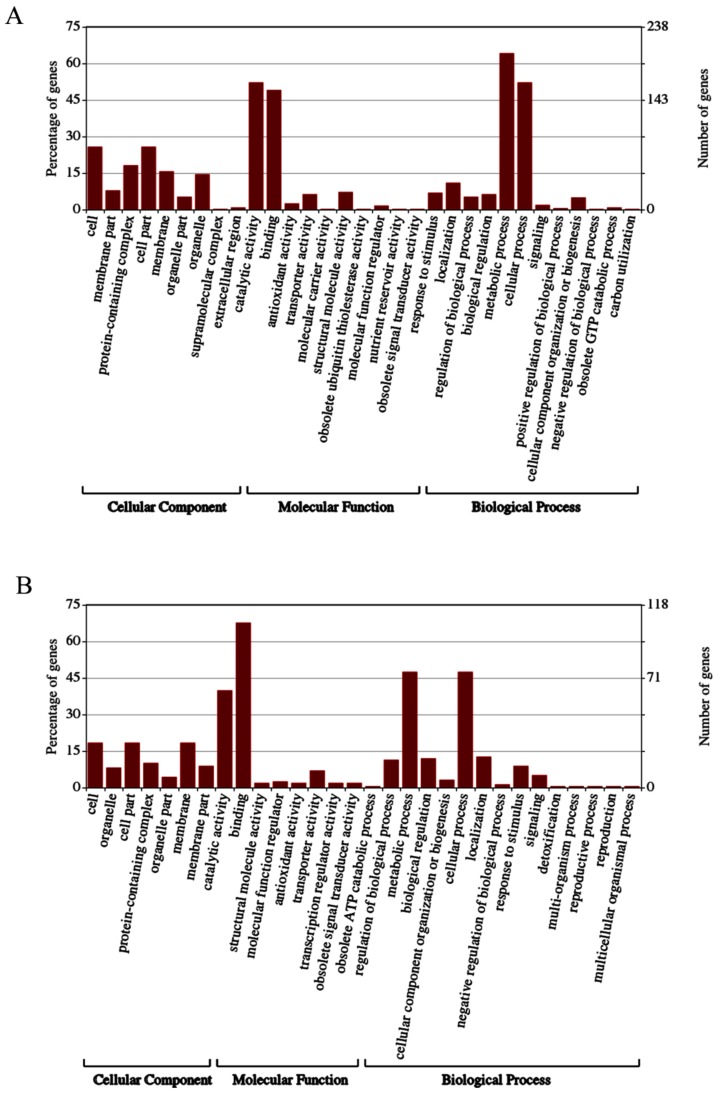
Gene ontology (GO) analysis of SEPs (**A**) and SCPL proteins (**B**). (**A**) The cellular components, biological processes, and molecular functions of differentially expressed proteins in samples; (**B**) The cellular components, biological processes, and molecular functions of differentially expressed phosphoproteins in samples.

WEGO is an useful tool for plotting GO annotation results, and it was widely used in many important biological research projects, such as the rice genome project [32] and the silkworm genome project [33]. It is also one of the routine tools for downstream gene annotation analysis, especially for comparative genomics tasks. To obtain a common GO classification for the identified proteins and phosphoproteins, functional analysis was conducted using WEGO annotation. Proteins and phosphoproteins were categorized according to their cellular components, molecular functions, and biological processes. As shown, proteins in 31 GO categories and phosphoproteins in 30 GO categories were affected by TMV infection (Figure 5A,B). 

Among the SEPs (Figure 5A), most proteins existed in the biological process category. Within this category, metabolic processes and cellular processes were highly represented, while the second most conspicuous GO categories were catalytic activity and binding in molecular functions; however, they were the most overrepresented GO categories among the SCPL proteins (Figure 5B). Apparently, the molecular functions of SCPL proteins were affected greatly by TMV infection. Metabolic processes and cellular processes under biological processes also accounted for a great proportion of the SCPL proteins. The cellular component of SEP and SCPL proteins was not significantly affected by TMV infection.

### 3.5. Motif Analysis

**Figure 6:**
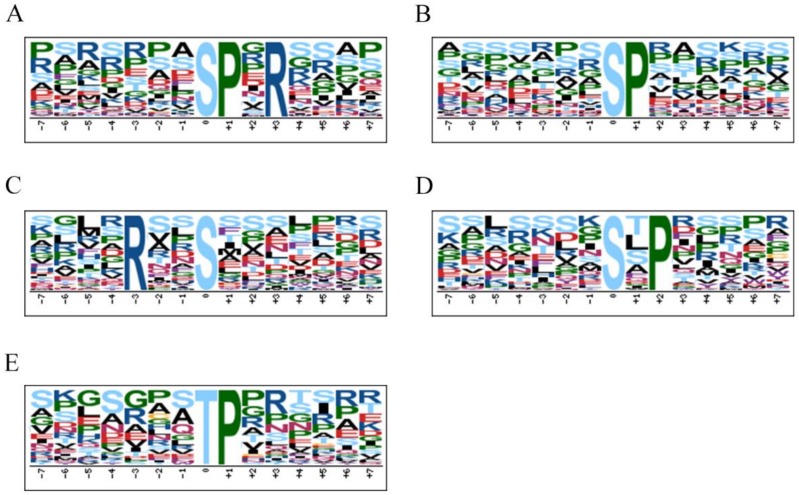
Phosphorylation motifs extracted from the overrepresented phosphopeptide dataset by Motif-X. (**A**)–(**D**) Five enriched motifs from phosphoserine; (**E**) Enriched motif from phosphothreonine.

Motif-X on-line tools were used to analyze the phosphorylation motifs of the phosphoproteins. As shown in Figure 6, Motif-X showed five motifs with accurate parameters (*p* < 10^−6^). In the T group, the overrepresented motifs obtained by enriching Ser and Thr were [sPxR], [sP], [Rxxs], [sxP], and [tP].

The basic motif [sPxR] (Motif A) is recognized by growth-associated histone kinase (GHK), cyclin-dependent kinase (CDK), or cell division cycle 2 (CDC2) kinase [7]. Most proteins containing [sP] (Motif B) are predominantly located in the nucleus and cytoplasm, and the proline-directed motifs are potential substrates of mitogen-activated protein kinase (MAPK), CDK, and CDK-like kinase [31,34]. The motif [Rxxs] (Motif C) is another common motif in plants and can be identified by mitogen-activated protein kinase kinase (MAPKK), calmodulin-dependent protein kinase (CaMK) II, and protein kinase A [31,34]. However, there is little information on the motif [sxP] (Motif D). The motif [tP] (Motif E) is the most common phosphothreonine motif found in plants, which is consistent with some previous reports about plant resistance [35,36].

### 3.6. PPI Analysis of Phosphoproteins

To examine the interactions of different protein kinases with their potential substrates, 338 identified SCPL proteins were analyzed using STRING. A total of 100 items were used to construct the PPI network. Cytoscape software was used to reconstruct the interaction network and displayed only the PPI network between certain known phosphoproteins, protein kinases/phosphatases, and five other important functional categories. To improve the reliability of PPI analysis, the confidence score was set to a high confidence level (>0.700). 

**Figure 7:**
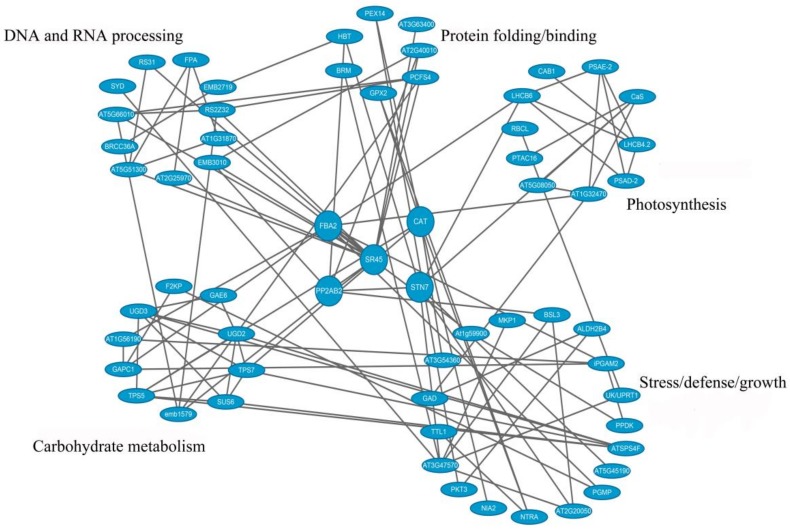
Protein–protein interaction networks of some important phosphoproteins in five functional categories.

As shown in Figure 7, items containing serine/arginine-rich 45 (SR45), protein phosphatase 2A (PP2A), catalase 2 (CAT), serine/threonine protein kinase (STN7), and fructose-bisphosphate aldolase 2 (FBA2) were connected with each other in this PPI map. These findings indicate that these molecules may play central roles in protein substrate phosphorylation/dephosphorylation in plant resistance to TMV infection.

## 4. Discussion

### 4.1. Phosphorylated Protein Kinases and Phosphatases Associated with Stress

The reversible protein phosphorylation/dephosphorylation process is regulated by protein kinases and phosphatases [37,38]. It plays major roles in multiple signal transduction processes. In this study, some protein kinases and phosphatases were SCPL proteins and were, thus, identified as crucial factors in the signal transduction responses to TMV infection.

Signaling by the phytohormone ABA with PP2C plays roles in the response to both abiotic stress and biotic stress [39,40,41]. Here, the PP2C protein was found among upregulated SCPL proteins involving the MAPK signaling pathway. Some studies noted that PP2C plays a positive regulatory role in the process of ABA signaling, along with ABA receptors (pyrabactin resistance 1 (PYR1)-like proteins (PYLs)) [42,43]. ABA signal transduction is elaborated by the interaction of PP2C and ABA receptors. PP2C-mediated ABA response mechanisms induce the inactivation of PP2C and relieve the inhibition of sucrose non-fermenting 1 (SNF1)-related protein kinases (SnRK). In drought conditions, the phosphorylation of PP2C might promote binding to PYLs and decrease the inhibition of SnRK activation to trigger the ABA signaling cascade [44]. Thus, the up-phosphorylated PP2C probably promotes the initiation of ABA-dependent signaling by binding to ABA receptors in tobacco to defend against TMV infection. This hypothesis is supported by similar results in rice, in which PP2C phosphorylation was upregulated by biotic stress [36].

Phosphoglycerate kinase (PGK) converts 1,3-bisphosphoglycerate into 3-phosphoglycerate in gluconeogenesis but also plays a key role in the Calvin–Benson cycle. PGK appears to be a potential candidate for increased salinity stress tolerance and enhanced yield in crop plants [45]. In addition, the chloroplast phosphoglycerate kinase (chl-PGK) from *Nicotiana benthamiana* is one of the viral RNA binding proteins involved in the Bamboo mosaic virus (BaMV) infection cycle. Chl-PGK might be involved in viral RNA localization in the chloroplasts [46,47,48]. In this study, the treatment samples contained several-fold more chl-PGK than the control tobacco without TMV infection. TMV is a single-stranded RNA virus, as is BaMV, and the mechanisms of some key regulators are similar. There is also a report about PGK that was reduced in TMV-infected plants after 10 days of inoculation [49]. It suggests that immediate PTM may take place after 48-h perturbation. Therefore, PGK could also be a potential candidate for increasing TMV infection tolerance in tobacco.

Pyruvate phosphate dikinase (PPDK) is known to be a key enzyme in gluconeogenesis and photosynthesis, and is responsible for reversing the reaction performed by pyruvate kinase in Embden–Meyerhof–Parnas glycolysis. It belongs to the family of transferases. PPDK was identified among the upregulated SCPL proteins in our study. Our findings are consistent with the previous report that PPDK plays a crucial role in plant resistance [50]. In particular, PPDK plays a controlling role in the phosphoenol pyruvate (PEP) regeneration phase of the C4 photosynthetic pathway [51]. It might improve the ability of *Arabidopsis thaliana* to resist drought stress, and the co-expression of PEPC and PPDK has a synergistic effect on stress tolerance [52]. Abiotic stresses such as gradual drying, cold, osmotic stress, and salt stress trigger a specific PPDK induction [53,54,55]. Thus, PPDK is a potential factor in plant resistance to TMV infection.

There are several differentially phosphorylated kinases or phosphatases, such as glutamate decarboxylase (GAD) and uridine diphosphate (UDP)-glucose 6-dehydrogenase, suggesting that the mechanism of tobacco defense against TMV infection is a highly complex event that involves multiple signaling pathways.

### 4.2. Translation Initiation and Transcription Factors

Many defense-related genes regulate signaling cascades via initiation and transcription factors. In this study, several initiation and transcription factors were identified among the differentially expressed proteins, such as eukaryotic translation initiation factor 4G (eIF4G), WRKY, and basic leucine zipper (bZIP).

Factor eIF4G plays a major role in initiation and provides a scaffold for ribosome/messenger RNA (mRNA)-bridging. It also regulates the recruitment of additional initiation factors [56]. It forms part of the protein complex eIF4F and participates in the recognition of the mRNA cap, recruitment of mRNA to the ribosome, and ATP-dependent unwinding of the 5′-terminal secondary structure. It also plays a crucial role in the accumulation of some potyviruses during viral infection [57]. These proteins are necessary for cell-to-cell virus movement [58]. Reducing eIF4G expression after development can increase lifespan, while its overexpression is involved in the malignant transformation of cells [59,60,61]. In brief, eIF4G overexpression could repress the symptoms of TMV-infected tobacco. WRKY participates in plant–pathogen interaction. Some cases of WRKY overexpression in other plant species prompt enhanced pathogen resistance by directly phosphorylating the transcription factor OsWRKY33 [62,63]. However, DNA-binding WRKY was downregulated after 48 h of TMV infection in tobacco, indicating that WRKY may be involved in the early response to TMV infection. Moreover, a bZIP domain-containing transcription factor RF2b was specifically dephosphorylated after TMV infection. Transcription factor RF2a, together with RF2b, was reported to suppress the replication of Rice tungro bacilliform virus (RTBV) by directly binding to its *cis*-element box II in the promoter. The disease symptoms may indicate that infection by RTBV reduces the availability of RF2a and RF2b [64,65,66]. In conclusion, bZIP plays a key role in TMV resistance and shows decreased availability after 48 h of TMV infection. These factors were found to be differentially phosphorylated, which suggests that they are good candidate proteins for TMV infection resistance in tobacco.

### 4.3. Tobacco Disease Resistance-Related Phosphoproteins

A phosphoprotein of RNA recognition motif domain (RRMs) was identified in the K326 database. Furthermore, nucleotide binding also exhibited a significantly upregulated phosphorylation level. For further analysis, the sequence of RRMs and nucleotide-binding proteins were identified in *Arabidopsis thaliana* database using the Basic Local Alignment Search Tool (BLAST). The result suggested that they were immensely similar to the sequence of SR45 and serine/arginine-rich splicing factor (SRSF) in *Arabidopsis thaliana*. Plant SR45 involved serine/arginine-rich (SR)-like RNA binding proteins [67]; SR45 is a member of the highly conserved family of serine/arginine-rich (SR) proteins, which play key roles in splicing events. SR45 is a key regulator involved in basal resistance by inducing the ABA signaling network or mRNA processing [68,69]. The SRSF is a cellular alternative splicing factor and a strong inhibitor of viral replication [70,71]. Some reports indicate that expression of AdRSZ21 with a mutated zinc knuckle (ZnK) domain leads to accelerated cell death [72,73]. Craigie et al. observed that SRSF1 inhibits ohn Cunningham virus (JCV) gene expression and viral replication by directly interacting with viral promoter sequences [74]. Though the reports were mostly in humans, the SRSF inhibition mechanism could be generalized to plants for TMV infection. To summarize, SRSF is developmentally regulated and modulated during virus infection [75].

Another differentially phosphorylated protein gene was a motor protein in K326. However, the motor protein had little specific information with the resistance of plant. Using BLAST analysis, it was found to be similar to the sequence of geminivirus Rep-interacting protein in *Arabidopsis thaliana*. Firstly, geminiviruses are small DNA viruses that replicate in the nuclei of infected plant cells after the accumulation of the host replication machinery [76]. In addition, reports suggested that Rep is essential for the replication of viruses such as tomato golden mosaic virus (TGMV) and tomato yellow leaf curl Sardinia virus (TYLCSV) [77]. Rep is an oligomeric protein that binds to double-stranded DNA, catalyzes the cleavage and ligation of single-stranded DNA, and is sufficient for host induction. It also interacts with several host proteins, including the cell-cycle regulator retinoblastoma protein and essential components of the cell DNA replication machinery [78]. Reyes proposed the use of Rep-binding peptide aptamers to develop crops that are resistant to geminiviruses [79]. According to our data, geminivirus Rep-interacting motor protein was upregulated in response to TMV infection, suggesting that Rep-interacting protein may play a crucial role in virus disease resistance. However, whether Rep-interacting protein could mediate resistance to TMV infection in tobacco or not needs to be further studied by genetic analysis and pathogen inoculation assays.

Autophagy is a positive response via which cells cope with endogenous or exogenous stress in unfavorable environments via an intracellular degradation pathway that is regulated by the autophagy-related (ATG) proteins. Autophagy is also an essential component of host immunity and is used by viruses for survival [80]. A surprisingly high number of ATG proteins (36%) have a positive or negative role in promoting virus replication outside their classical role in autophagy [81]. TMV induces autophagosome formation by inducing endoplasmic reticulum stress, and some data indicate that HeLa cells use endoplasmic reticulum stress (ERS) and ERS-related autophagy to defend against TMV RNA [82]. Atg13 provides inter-complex connections between the Atg17/Atg29/Atg31 complexes, thereby accelerating the initial events of autophagy, including the autophosphorylation of Atg1, recruitment of Atg9 vesicles, and phosphorylation of Atg9 by Atg1 [83]. Autophagy-related protein 13 (ATG13) was identified in both cultivars and showed upregulated phosphorylation levels compared to the control. Our evidence indicates that ATG13 may play an important part in tobacco TMV resistance.

In the face of various biotic and abiotic stresses, particularly TMV infection, phytohormones gradually adjust the plant growth mechanisms. As described above with regards to ABA signaling with protein PP2C, salicylic acid (SA), indole-3-acetic acid, and auxin were the limiting factors in plant immunity responses. Auxin also affects SA signaling and modulates disease resistance [84,85]. Ghanashyam et al. provided evidence for the role of auxin in plant defense responses and suggested cross-talk among the auxin, abiotic stress, and biotic stress signaling pathways [86]. In our study, dormancy/auxin-associated protein was identified among the upregulated SPLC proteins, which includes the auxin-repressed 12.5-kDa protein (ARP). There were also some reports about the involvement of ARP in plant growth and disease resistance. In tobacco, ARP is encoded by an isolated *GERI* gene and is essential for salicylic acid-mediated defense. Using genetic complementation, gene silencing, and overexpression analyses, this gene was further characterized as an activator of disease resistance and a repressor of plant growth that acts by inhibiting the expression of auxin response factor gene *ARF8* [87,88,89]. The defense response gene *GERI* showed repressed expression, which is consistent with impaired resistance to diseases caused by viruses, bacteria, and other pathogens.

### 4.4. Proteins Involved in Disease

The SEPs included some proteins with high-fold change accumulation, such as thaumatin; thaumatins are structurally diverse and are ubiquitously associated with osmotic adaptation in plant cells. Osmotin and osmotin-like protein (OLP) are homologous to the thaumatin protein [90,91]. Some results provide evidence that a potato osmotin-like protein (OLP) has antifungal activity against the fungus *Phytophthora infestans* [92]. Hence, thaumatin and OLP are certainly related to plant disease. The overexpression of these proteins was induced by TMV infection and wounding. The accumulation of osmotin mRNA was also reported to occur in some plant tissues in response to treatment with ABA, wounding, and TMV infection, which was controlled developmentally by at least five hormonal or environmental signals. However, post-transcriptional processes can limit osmotin accumulation [93]. OLPs mediate defense against abiotic and biotic stresses in plants. The overexpression of an OLP gene in plants enhances tolerance against drought, salinity, oxidative stress, and the charcoal rot pathogen [94]. The expression patterns of plant defense genes encoding osmotin and OLPs imply an important function in osmotic stress and plant pathogen defense.

## 5. Conclusions

In this study, the first phosphoproteomic analysis of TMV-infected tobacco was performed. A total of 1148 phosphoproteins with 3063 phosphorylation sites were detected in tobacco using iTRAQ labeling quantification. The analysis of SEPs and SCPL proteins in the TMV-infected tobacco revealed that 277 proteins were differentially phosphorylated, including several well-known tobacco disease resistance-related proteins, such as SR45, PGK, PP2C, PPDK, and ATG13. These proteins might shed new light on the roles of phosphorylation in plant disease resistance. Our results extend the current knowledge of TMV-infected tobacco and its viral defense mechanisms from the perspective of phosphoproteomics.

## Supplementary Materials

The following are available online at http://www.mdpi.com/2218-273X/9/2/39/s1: Table S1: List of all identified specifically expressed proteins (SEPs) of total protein from tobacco (*Nicotiana tabacum*); Table S2: List of all identified significant changes in phosphorylation level (SCPL) proteins of total phosphoprotein from tobacco (*Nicotiana tabacum*); Table S3: List of all identified differential components by GC–MS from tobacco (*Nicotiana tabacum*); Figure S1: Workflow of quantitative phosphoproteomic analysis in two K326 cultivar groups; Supplemental S1: The genome location of TMV primer hybridization.
